# Epileptic encephalopathy in a young Bengal cat caused by CAD deficiency

**DOI:** 10.1038/s41598-025-98414-0

**Published:** 2025-04-18

**Authors:** Adriana Kaczmarska, Matthias Christen, Francisco del Caño-Ochoa, Santiago Ramon-Maiques, Ana Cloquell Miro, Angie Rupp, Vidhya Jagannathan, Tosso Leeb, Rodrigo Gutierrez-Quintana

**Affiliations:** 1 School of Biodiversity, One Health and Veterinary Medicine, University of Glasgow, Glasgow, G61 1QH UK; 2 Vetsuisse Faculty, Institute of Genetics, University of Bern, Bremgartenstrasse 109a, 3001 Bern, Switzerland; 3 Instituto de Biomedicina de Valencia (IBV), CSIC, Valencia, Spain; 4 Group CB06/07/0077, Centro de Investigación Biomédica en Red de Enfermedades Raras (CIBERER)–Instituto de Salud Carlos III, Valencia, Spain; 5 Valencia Biomedical Research Foundation, Centro de Investigación Príncipe Felipe (CIPF) - Associated Unit to the Instituto de Biomedicina de Valencia (IBV), Valencia, Spain

**Keywords:** Felis catus, Neurogenetics, Developmental and epileptic encephalopathy, Precision medicine, Animal model, Experimental models of disease, Genetics research, Disease genetics, Neurological disorders, Metabolic disorders, Epilepsy, Agricultural genetics

## Abstract

Developmental and epileptic encephalopathy type 50 (DEE50) in humans is a severe early-onset neurometabolic disorder caused by biallelic loss-of-function variants in the *CAD* gene encoding a key multi-enzymatic protein for de novo pyrimidine nucleotide synthesis. Untreated, the condition is often fatal, but patients respond to uridine supplementation, which fuels nucleotide synthesis through CAD-independent salvage pathways. Here, we report a novel variant in the feline *CAD* gene in a 4-month-old Bengal kitten with intractable seizures and abnormal behavior. The variant, XP_011279586.1:p.(Ser2015Asn), was predicted to affect the oligomerization of the C-terminal aspartate transcarbamylase (ATCase) domain of CAD. Genotyping of 110 unaffected Bengal cats revealed four additional carriers of the mutant allele, confirming its presence in the breed. In a CAD-knockout human cell line dependent on uridine, the recombinant expression of human wildtype CAD, but not of the Asn2015 mutant, restored cell growth without uridine, demonstrating that the p.Ser2015Asn variant disrupts CAD function and is pathogenic. This study facilitates genetic testing of carriers and affected cats and suggests that uridine supplementation could be a potential treatment. Furthermore, CAD-deficient Bengal cats might serve as a valuable spontaneous large animal model to further investigate the pathogenic mechanisms of this rare epileptic encephalopathy in humans.

**Supplementary Information:**

The online version contains supplementary material available at 10.1038/s41598-025-98414-0.

## Introduction

The *CAD* gene codes for carbamoyl-phosphate synthetase 2, aspartate transcarbamylase, and dihydroorotase, a multi-enzymatic protein that catalyzes the first steps of de novo biosynthesis of uridine monophosphate (UMP) and other pyrimidine nucleotides and regulates the flux through the pathway^1,2^. Pyrimidine nucleotides are essential building blocks of nucleic acids and play an important role in protein glycosylation, lipid metabolism, polysaccharide biosynthesis, and signal transduction^3,5^. Biallelic pathogenic variants in the *CAD* gene cause autosomal recessive developmental and epileptic encephalopathy type 50 (DEE50) in human patients (OMIM#616457)^3,6,7^. This form of epileptic encephalopathy often presents with anemia and anisopoikilocytosis and leads to cerebral and cerebellar atrophy. Although if untreated, this neurometabolic disorder is often fatal, patients respond effectively to oral supplementation of uridine (UMP or uridine triacetate), which fuels pyrimidine synthesis through CAD-independent salvage pathways. No side effects are reported for uridine supplementation, which is recommended for cases of intractable epilepsy and developmental delay of unknown origin^3^. Since the first case in 2015^6^, about 50 CAD-deficient patients have been reported worldwide. Although the condition seems rare, it is likely underdiagnosed due to overlapping symptoms with other disorders, the absence of a specific biomarker^3,8,9^ and the presence of over 2,400 CAD missense variants of unknown pathogenicity carried among the healthy population (gnomAD v4.1.0; https://gnomad.broadinstitute.org).^8,10^

Here, we report the first case of CAD deficiency in a cat. We identified a novel pathogenic variant in the feline *CAD* gene, XP_011279586.1:p.(Ser2015Asn), in a 4-month-old Bengal kitten with intractable seizures and abnormal behavior. The affected residue is conserved in human CAD, although this particular feline variant has not been observed in affected children so far. Using a functional cell proliferation assay, we demonstrate that the p.Ser2015Asn variant disrupts human CAD activity and confirmed its pathogenicity.

## Materials and methods

### Ethics statement

All examinations and animal experiments were carried out after obtaining written informed owners’ consent and in accordance with local laws, regulations, and ethical guidelines. Investigations on the affected cat were performed as part of veterinary treatment and diagnostics and did not constitute an animal experiment in the legal sense. The study obtained the approval of the Cantonal Committee for Animal Experiments (Canton of Bern, Switzerland; permit BE94/2022).

### Clinical investigations

Investigations were performed as part of the clinical work-up for this cat, and the owners signed a consent form allowing the use of data for clinical research. The following data were obtained: signalment, clinical history, details of physical and neurological examinations, treatment and outcome. The diagnostic workup included routine hematology, serum biochemistry, ammonia level, serology for infectious diseases (*Toxoplasma gondii*, Feline Immunodeficiency Virus (FIV), and Feline Leukemia Virus (FeLV)) and auto-immune limbic encephalitis (serum feline-specific antibodies to leucine-rich glioma-inactivated 1 (LGI1)), magnetic resonance imaging (MRI) of the brain with a 1.5 T scanner (Magnetom, Essenza, Siemens Healthineers, Forcheim, Germany), cerebrospinal fluid (CSF) analysis, and screening for organic acidurias. Samples from dam, sire or littermates could not be obtained.

### Genetic investigation

Genomic DNA was extracted from EDTA blood samples using the Maxwell RSC Whole Blood Kit and the Maxwell RSC instrument (Promega, Dübendorf, Switzerland). An Illumina TruSeq DNA PCR-free library from the affected cat was prepared and 235 million 2 × 150 bp paired-end reads were collected on an Illumina NovaSeq 6000 instrument corresponding to 27.3 × coverage (Illumina, Zürich, Switzerland). Alignment to the F.catus_Fca126_mat1.0 reference genome assembly and variant calling were performed as previously described^11^. The sequence data were submitted to the European Nucleotide Archive under the study accession PRJEB7401 and the sample accession SAMEA14502952. Additionally, genome sequence data from 99 control cats were included in the analysis (Table S1). To predict the functional effects of the called variants, SnpEff software^12^ together with the F.catus_Fca126_mat1.0 reference genome assembly and NCBI annotation release 105 was used. The bioinformatics analysis identified a single homozygous private missense variant in the functional candidate gene *CAD.* The candidate variant was independently confirmed and genotyped by direct Sanger sequencing of PCR amplicons. Primers 5’-TTCCCCAAGATGAACACACA-3’ and 5’-TGCCTGAGTCCCACTCTTCT-3’ were used for the generation of an amplicon containing the CAD: XM_011281284.3:c.6044 C > T variant. PCR products were amplified from genomic DNA using AmpliTaq Gold 360 Master Mix (Thermo Fisher Scientific, Reinach, Switzerland). Direct Sanger sequencing of the PCR amplicons on an ABI 3730 DNA Analyzer (Thermo Fisher Scientific) was performed after treatment with exonuclease I and alkaline phosphatase. Sanger sequences were analyzed using the Sequencher 5.1 software (Gene Codes, Ann Arbor, MI, USA). Additionally, 110 unaffected Bengal cats were genotyped.

## In silico prediction of pathogenicity

Pathogenicity prediction was performed with AlphaMissense^13^ and FoldX^14^. AlphaMissense scores and pathogenicity predictions were retrieved from https://github.com/google-deepmind/alphamissense. FoldX 5.0 was run locally using default settings and the models of the crystallographic ATC (PDB 5G1O; 2.1 Å resolution). Before performing calculations, the ‘RepairPDB’ command was used to fix the structure. Individual mutations were introduced using the ‘BuildModel’ command. Calculations were performed in 5 consecutive runs, and ΔΔG values were extracted from the FoldX output files. A value of 1.5 kcal/mol was set as the stability change threshold, as previously suggested in other studies assessing the impact of missense variants^14^.

## Functional analysis

The functional cell assay was performed as detailed by Del Caño-Ochoa et al. 2020^8^. In brief, the functional effect of the variant was assessed using a human CRISPR/Cas9 edited *CAD*-knockout cell line that requires uridine supplements for survival. The cells were transiently transfected with a cDNA encoding wild-type or Asn2015 mutant human CAD, fused at the N-terminus to the green fluorescent protein (GFP) and proliferation was monitored over a week in media without uridine. Normal cell growth suggests that the variant is likely benign, while impaired proliferation indicates that the variant affects CAD activity and is pathogenic.

## Results

### Clinical data

A 4-month-old male Bengal kitten was presented with a three-week history of clusters of generalized tonic seizures with orofacial involvement and abnormal behavior. The owners reported the first seizure at 13 weeks of age. The seizures occurred during sleep and were characterized by sudden jumps, followed by opisthotonus associated with increased muscle tone of the thoracic limbs, head swaying, lip smacking, chewing movements, facial twitches, salivation and impaired consciousness (Video S1). The seizures lasted a few seconds to a minute, and afterwards, the cat was disorientated. The kitten was in general quieter than a healthy kitten of its age. Additionally, the cat displayed multiple episodes of abnormal behavior manifested as events of obtundation or agitation, occasionally accompanied by biting the floor and its own paws. The kitten experienced multiple episodes of cluster seizures but never progressed to status epilepticus.

General physical examination revealed mild ocular discharge and a good body condition (score of 4/9) with a weight of 1.67 kg. Neurological examination was normal except for episodes of abnormal mentation and absent menace response bilaterally.

Results of blood work (hematology, biochemistry, ammonia) were normal except for mild lymphocytosis 9.7 × 10^9^/L (reference range: 0.98–6.88 × 10^9^/L) and mildly increased red cell distribution width (RDW) 28.7% (reference range: 15–27%) which may indicate mild anisocytosis. Further diagnostic tests, which comprised high-field 1.5 T MRI of the brain, CSF analysis, and urine organic acids, were largely unremarkable. Serology for infectious diseases, including *Toxoplasma gondii*, FeLV and FIV, as well as for autoimmune limbic encephalitis (feline anit-LGI1 antibodies) was negative. The kitten showed only partial response to treatment with phenobarbital (2.4 mg/kg BID) and levetiracetam (20 mg/kg TID). Therefore, the owners elected euthanasia due to the impaired quality of life of the kitten. The results of the ancillary tests are provided in the supplementary materials (Table S2).

## Genetic analysis

**Fig. 1:**
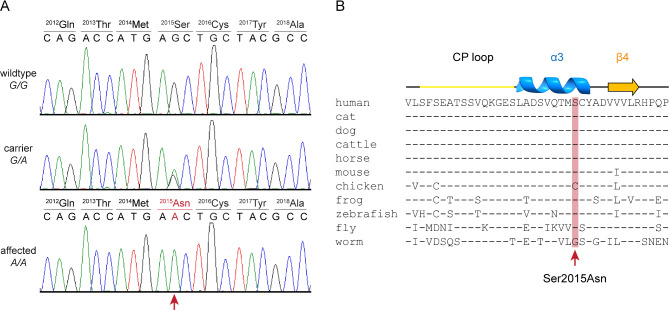
Details of the XP_006930506.2:p.(Ser2015Asn) variant are presented. (**A**) Sanger electropherograms derived from the genomic DNA of a wildtype control, a carrier, and the affected cat, highlighting the presence of the Ser2015Asn variant. The electropherograms show the wildtype (G/G), carrier (G/A), and affected (A/A) genotypes at the XM_011281284.3:c.6044G>A variant. (**B**) A multiple species alignment shows the high evolutionary sequence conservation surrounding the Ser2015Asn variant. The secondary structure motifs are indicated, with the α3 helix and β4 sheet highlighted to provide structural context for the position of the variant.

As an inherited disease was suspected, the genome of the affected kitten was sequenced, and the data was compared to 99 control cat genomes. This analysis yielded 19 homozygous and 1407 heterozygous private protein-changing variants (Table S3). The variants were prioritized based on the known functions of the affected genes. The top candidate causal variant for the observed phenotype was ChrA3:NC_058370.1:g.118,599,900 C>T, a private homozygous missense variant in the *CAD* gene, XM_011281284.3:c.6044G>A corresponding to XP_011279586.1:p.(Ser2015Asn) at the protein level (Fig. 1). Genotyping of 110 unaffected Bengal cats identified 4 additional carriers, confirming the presence of the mutant allele at low frequency in the breed. None of the unaffected cats carried the mutant allele in a homozygous state.

### In silico and functional genomic analyses

**Fig. 2:**
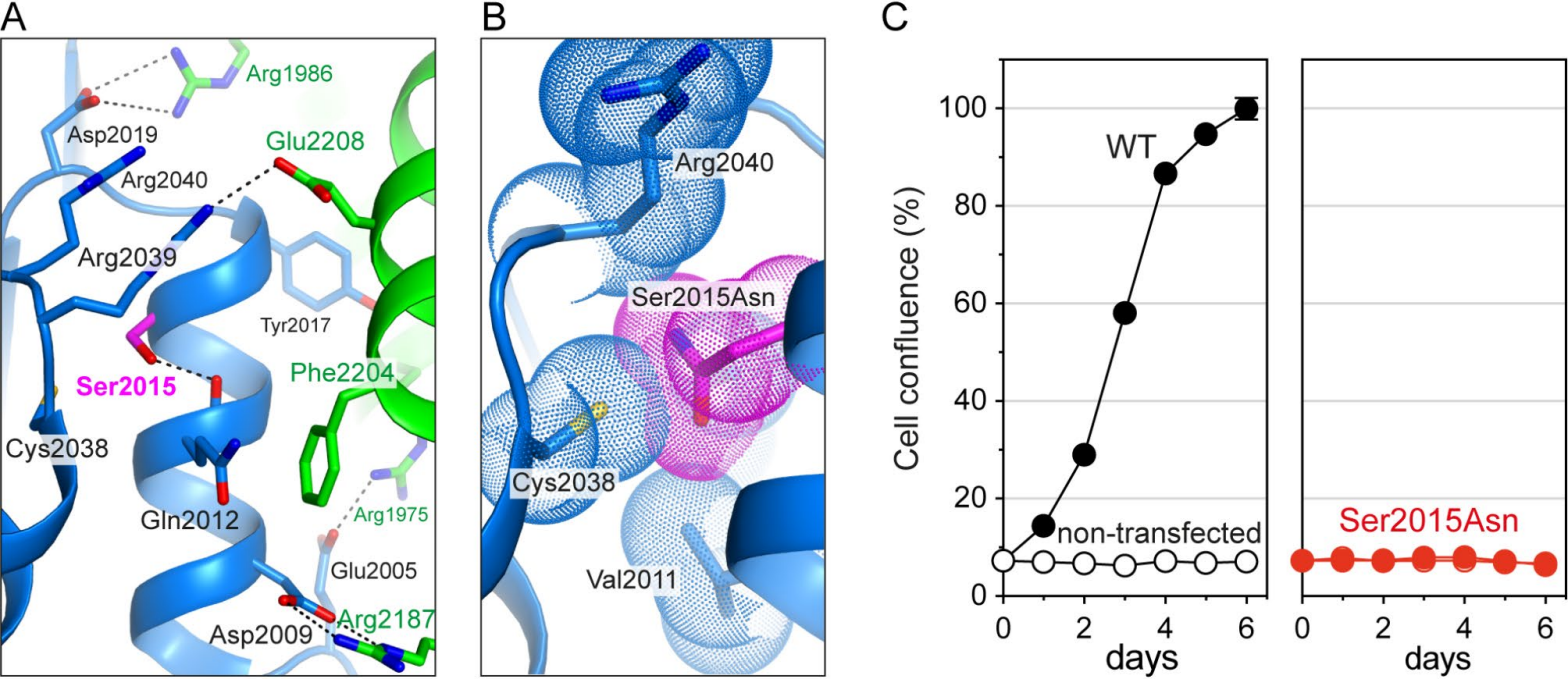
Structural and functional impact based on in silico and functional genomics analyses of the human CAD variant p.Ser2015Asn. (**A**) Cartoon representation of human CAD’s ATC structure (PDB accession code 5g1n) is shown forming a homotrimer. The side chain of Ser2015 is depicted in magenta, with neighboring subunits represented in blue and green. Electrostatic interactions are indicated with dashed lines. (**B**) Molecular modeling of the Ser2015Asn variant shows steric clashes with neighboring residues. The atomic radii are represented as dot clouds. (**C**) Cell proliferation assay of CAD-knockout cells transfected with expression constructs for either wildtype (WT) or Ser2015Asn mutant human CAD. WT cells (black dots) show normal growth, while cells with the Asn2015 mutant (red dots) fail to grow over six days, indicating impaired CAD function.

The human CAD protein (NP_00433.2) and feline CAD protein (XP_011279586.1) are both 2,225 amino acids long and share 98% identical residues. The affected residue, Ser2015, is highly conserved across animals and is located in the aspartate transcarbamylase (ATC) domain of CAD. The crystal structure of this domain^15^ shows that this Ser is at the C-terminus of an α-helix critical for oligomerizing the ATC into a homotrimer (Fig. 2A). The Ser side chain forms a hydrogen bond within the helix and Van der Waals interactions with Cys2038 and Arg2040 in an adjacent loop. Replacing Ser with Asn introduces a larger side chain, causing steric clashes that are predicted to affect stability and folding and impair oligomerization of ATC, which in turn is critical for correct CAD function (Fig. 2B).

We also used computational tools to predict the damaging potential of the variant. AlphaMissense^13^ trained on population frequency data and utilizing sequence information and AlphaFold2-predicted structures, classified Ser2015Asn as ambiguous, with a score of 0.3822. Additionally, we employed FoldX^14^ to estimate the change in Gibbs free energy (ΔΔG) of folding for the protein before and after a mutation. A large positive ΔΔG (typically > 1.5 kcal/mol) suggests that the mutation reduces protein stability and is therefore considered as an indicator of pathogenicity. Using the ATC homotrimer structure, FoldX predicted ΔΔG values of 4.71 ± 0.55, 5.03 ± 0.41 and 3.4 ± 0.18 for the three subunits, indicating that mutation Ser2015Asn is destabilizing and likely pathogenic.

To assess the pathogenicity of the variant in a functional experiment, we performed a cellular growth complementation assay using a CAD-knockout (KO) cell line dependent on uridine for survival^6^. This assay identifies variants that impair CAD activity as these result in a lack of cell growth without uridine supplementation, thus indicating pathogenicity, while normal growth suggests the variant is likely benign. To date, this assay remains the most reliable tool for assessing the functionality of CAD variants and has been instrumental in diagnosing CAD-deficient patients worldwide^8,16^. Thus, CAD-KO cells were transfected with plasmids encoding either wild-type (WT) or the Asn2015 mutant human CAD, and proliferation was monitored for a week after uridine removal. Cells expressing WT CAD restored pyrimidine synthesis and proliferated normally, while those expressing the Asn2015 mutant failed to proliferate, confirming the pathogenicity of the p.Ser2015Asn variant (Fig. 2C).

## Discussion

Here, we present a case of a Bengal kitten with epileptic encephalopathy associated with CAD deficiency, caused by the pathogenic variant p.Ser2015Asn. CAD is a 2,225 amino acids long protein highly conserved in animals that evolved by the fusion of four enzymatic domains, each catalyzing one of the initial steps in de novo pyrimidine nucleotide synthesis: glutamine amidotransferase (GAT), carbamyl phosphate synthetase II (CPS2), aspartate transcarbamylase (ATC), and dihydroorotase (DHO)^2,17^. Since its discovery, CAD has been known to oligomerize, primarily forming hexamers about half the size of a ribosome^18,19^. Recent studies on pathogenic variants in CAD-deficient human patients have confirmed that this assembly, which critically depends on the oligomerization through the ATC domain, is essential for CAD’s proper function^20^.

The ATC domain self-assembles into a homotrimer, which has three active sites at interfaces of the subunits. Thus, ATC is only active as a trimer, and variants that disrupt oligomerization inactivate the enzyme^20,21^. The reported Ser2015Asn variant is predicted to alter the folding and stability of a key α-helix for ATC oligomerization. The helix follows a loop that completes the active site in the adjacent subunit of the trimer and binds to the substrate carbamoyl phosphate (CP-loop). This helix is surrounded by four ion pairs that maintain the subunits attached. Variants that impair some of these interactions have been shown to disrupt ATC oligomerization and thus, impair CAD activity^20,21^. Similarly, we propose that the pathogenic mechanism of the Ser2015Asn variant identified in this study is a reduction in the stability of the helix at the ATC interface, impairing oligomerization and enzymatic activity. FoldX calculations support this destabilizing effect, while AlphaMissense failed to detect the variant’s impact, likely because it uses AlphaFold2 models that do not account for protein oligomerization.

A CAD enzyme defect can be bypassed by the salvage of uridine, which is converted to UMP by the enzyme uridine cytidine kinase (UCK2). Most patients receiving uridine supplementation experience seizure cessation or significant reduction in seizure frequency and severity, resolution of hematological abnormalities and improvement in developmental status^3,7,22–29^. The efficacy of this treatment in the presented Bengal kitten could not be confirmed, as it was euthanized before receiving the genetic test results. However, based on the CAD-KO cell assay, there is no doubt that the variant impairs CAD function, and the kitten would likely have responded well to uridine therapy.

For a formal classification of variant pathogenicity according to the recently proposed animal variant classification guidelines (AVCG)^30^, we collected the following arguments: A well-established in vitro functional study supported the damaging effect on the gene product (PS3), computational evidence supported a deleterious effect on protein trimerization (PP3), and the patient’s phenotype was highly specific for a disease with single gene etiology (PP4). The combination of one strong and two supporting criteria allows to classify the variant as likely pathogenic. However, it has to be cautioned that the newly proposed AVCG have not yet been endorsed by an official body. In contrast to the corresponding human ACMG/AMP criteria^31^, they do not consider the absence of the homozygous mutant genotype from a large control cohort as supporting evidence. Furthermore, the PS3 criterion relating to the functional confirmation necessarily needs to take into account that different types of functional confirmation experiments may yield different levels of support for pathogenicity. We argue that the well-established CAD-KO cell assay is highly specific and sufficient to definitively prove the causality of the detected variant. Finally, neither ACMG/AMP nor AVCG classification guidelines adequately consider comparative insights from other species. The knowledge on the genetics and pathophysiology of human CAD deficiency corroborates the findings in the investigated cat. Thus, we think that in this case, the AVCG classification of likely pathogenic is overly conservative.

We report the first animal diagnosed with a CAD deficiency. Previously, a *CAD* missense variant, p.Tyr452Cys, was reported to be responsible for embryonic lethality with incomplete penetrance in French Normande cattle (OMIA:002201–9913) using a large-scale screening of homozygous haplotype deficiency. However, the only homozygous cow detected for this variant was not clinically studied and was only reported by the breeder to have poor udder development^32^.

The clinical phenotype of the Bengal kitten closely resembles that of children with DEE50^3,7,22–26^. The kitten presented with generalized tonic seizures and behavioral abnormalities, the latter of which may indicate developmental delay, both being hallmark features of DEE50. The episodes of abnormal behavior could also represent post-ictal changes or focal seizures. Other common signs in humans, such as ataxia, tremors, dysphagia, hypotonia, or strabismus, were not observed^23^. However, since the kitten was euthanized early in the disease course, these clinical signs may have manifested later.

The absence of a menace response could be post-ictal or due to developmental delay, as healthy cats develop this response at three months of age. Visual impairment, as seen in an 8-month-old CAD-deficient child could not categorically be ruled out either, however seemed less likely given the normal MRI findings^29^.

The kitten also displayed increased red cell distribution width (RDW) and possibly mild anisocytosis, features typical of CAD deficiency in humans, which tend to improve with uridine treatment^3,6,7,22–24^. This hematopoietic dysfunction likely stems from a lack of pyrimidine-dependent nucleotide-lipid cofactors essential for erythrocyte membrane synthesis^3,7,23^. However, a peripheral blood smear was not evaluated, therefore anisocytosis could not be confirmed and remains speculative.

MRI of the kitten’s brain showed no abnormalities, whereas in humans, MRI findings can range from normal to exhibiting cerebral, or more commonly cerebellar atrophy, with increased age correlating brain atrophy, especially in the cerebellum^3,24^. Increased susceptibility of the cerebellum may be due to persistent high CAD expression patterns in this tissue whilst the cerebrum exhibits a decline postnatally^22^. Cerebellar involvement in the kitten might have become evident with disease progression if it had survived longer. We acknowledge several limitations of our study, including the absence of a definitive histopathological diagnosis, the lack of electroencephalographic (EEG) assessment of the kitten’s seizures activity and not having performed a trial evaluating the response to uridine therapy due to the early euthanasia. Performing EEG in small animals, especially young kittens, presents technical challenges due to their size and limited tolerability of the equipment and is therefore rarely performed.

In conclusion, this is the first report of a naturally occurring CAD deficiency in animals that closely resembles DEE50 in humans. Cats with this variant could serve as a spontaneous large animal model to further explore the pathogenesis of this rare epileptic encephalopathy. Moreover, this study facilitates genetic testing to identify carriers and affected cats and proposes uridine supplementation as a potential treatment for affected kittens.

## Electronic supplementary material

Below is the link to the electronic supplementary material.

Supplementary Material 1

Supplementary Material 2

Supplementary Material 3

Supplementary Material 4

Supplementary Material 5

## Data Availability

The data supporting the findings of this paper are available in the supplementary materials. Whole genome sequence accessions are listed in Table S1.
