# Supplementary material for: Epileptic encephalopathy in a young Bengal cat caused by CAD deficiency
Source: Sci Rep. 2025 Apr 18;15:13506. doi: 10.1038/s41598-025-98414-0 (PMC12008240; doi:10.1038/s41598-025-98414-0)
Supplement: Supplementary file 5 — Supplementary Material 5 [file 41598_2025_98414_MOESM5_ESM.docx]

**Legend for Supplementary Video 1:**

**Video S1**: Recording of an epileptic seizure of the affected cat.
